# Improvement of Direct Interspecies Electron Transfer *via* Adding Conductive Materials in Anaerobic Digestion: Mechanisms, Performances, and Challenges

**DOI:** 10.3389/fmicb.2022.860749

**Published:** 2022-03-30

**Authors:** Le Chen, Wei Fang, Jianning Chang, Jinsong Liang, Panyue Zhang, Guangming Zhang

**Affiliations:** ^1^Beijing Key Lab for Source Control Technology of Water Pollution, College of Environmental Science and Engineering, Beijing Forestry University, Beijing, China; ^2^Engineering Research Center for Water Pollution Source Control and Eco-Remediation, College of Environmental Science and Engineering, Beijing Forestry University, Beijing, China; ^3^School of Energy and Environmental Engineering, Hebei University of Technology, Tianjin, China

**Keywords:** anaerobic digestion, direct interspecies electron transfer, conductive materials, methanogenesis, syntrophic metabolism

## Abstract

Anaerobic digestion is an effective and sustainable technology for resource utilization of organic wastes. Recently, adding conductive materials in anaerobic digestion to promote direct interspecies electron transfer (DIET) has become a hot topic, which enhances the syntrophic conversion of various organics to methane. This review comprehensively summarizes the recent findings of DIET mechanisms with different mediating ways. Meanwhile, the influence of DIET on anaerobic digestion performance and the underlying mechanisms of how DIET mediated by conductive materials influences the lag phase, methane production, and system stability are systematically explored. Furthermore, current challenges such as the unclear biological mechanisms, influences of non-DIET mechanisms, limitations of organic matters syntrophically oxidized by way of DIET, and problems in practical application of DIET mediated by conductive materials are discussed in detail. Finally, the future research directions for practical application of DIET are outlined.

## Introduction

Methane production from anaerobic digestion can reduce the dependence on fossil energy, which is critical to realize the current global sustainable development goals of climate change, waste recycling, and clean energy production (Wang P. et al., 2018; Li et al., 2019; Akor et al., 2021; Wang et al., 2021). Generally, four groups of microorganisms (fermentative bacteria, acidogens, acetogenes, and methanogens) with specific metabolic functions participate in anaerobic digestion process (Alavi-Borazjani et al., 2020; Abbas et al., 2021). These microorganisms have distinct characteristics on physiology, nutrient metabolism, growth kinetics, and environmental sensitivity (Luo et al., 2016). Thus, the interactions between microorganisms are complex during the entire anaerobic digestion process. Compared with methanogens, bacteria have a shorter generation time, faster growth rate, and more extensive adaptability to pH variations. The imbalance between methanogens and bacteria usually impairs anaerobic digestion performance (Feng et al., 2019). Hence, the efficient syntrophic associations between methanogens and bacteria play an important role in anaerobic digestion process.

For many years, interspecies hydrogen transfer (IHT) and interspecies formate transfer (IFT) are believed to be two common modes for interspecies electron transfer in syntrophic oxidation of organic matters (Dong and Stams, 1995; Zhao et al., 2016b). However, H_2_ production in syntrophic oxidation with energy consuming is an unfavorable thermodynamic reaction (Li Y. et al., 2018; Zhao J. et al., 2018). Recently, direct interspecies electron transfer (DIET) has been proved to be a new syntrophic association mode, in which electrons transfer directly from electron donors to electron acceptors with fewer biological enzymatic reactions (Summers et al., 2010; Gahlot et al., 2020). DIET can replace IHT in anaerobic digestion systems because it does not require H_2_ as an electron carrier, overcoming the thermodynamic limitation under high hydrogen partial pressure (Baek et al., 2018; Zhao and Zhang, 2019). A mathematical model shows that the electron transfer rate based on DIET was 8.57 times higher than that of IHT (Storck et al., 2015). Therefore, the electron transfer capacity of DIET is more efficient than that of IHT (Zhao et al., 2016a; Wang et al., 2020c; Zhang et al., 2020).

Currently, *Geobacter* species has been proven to be able to establish DIET-based syntrophic associations with methanogens *via* conductive pili (e-pili) and c-type cytochrome (OmcS), in which methanogens accept electrons and reduce CO_2_ to methane (Rotaru et al., 2014a,b). Studies have shown that various bacteria that cannot produce e-pili or OmcS can also build DIET-based syntrophic partnerships through adding conductive materials, such as granular activated carbon (GAC) (Zhang et al., 2020), biochar (Qi et al., 2021), carbon cloth (Jia et al., 2020), carbon nanotube (Shen et al., 2020), graphene (Igarashi et al., 2019), and magnetite (Cheng et al., 2020). It is generally agreed that non-biological conductive materials act as an electrical conduit to replace the function of e-pili or OmcS (Chen et al., 2014a; Xu et al., 2020).

The discovery of DIET provides a new way to improve anaerobic digestion performance. DIET mediated by conductive materials has been successfully applied to anaerobic digestion systems with different substrates over the years. As of late, several corresponding reviews have been published, elaborating the importance and advantages of DIET mediated by conductive materials (Barua and Dhar, 2017; Baek et al., 2018; Martins et al., 2018; Park et al., 2018a; Xu et al., 2019; Gahlot et al., 2020; Nguyen et al., 2021). However, there are few reviews on comprehensively summarizing the mechanisms of DIET and the effects of DIET mediated by conductive materials on anaerobic digestion performances. Furthermore, some significant questions need to be answered. For example, the biological path of electron transfer remains unclear (Yin and Wu, 2019). In addition to DIET, other stimulatory mechanisms of conductive materials also influence anaerobic digestion performances, such as microbial immobilization, buffering effect, and adsorption effect, which are related to the surface physicochemical characteristics of conductive materials, such as large surface area, rich redox groups and surface pH (Martins et al., 2018; Park et al., 2018a). These elements of knowledge should be considered because it is difficult to determine the contribution of non-DIET function to methane production. Meanwhile, limited substrates and microorganisms have been proved to be involved in DIET, and some substrates and microorganisms are only speculated to be involved in DIET according to anaerobic digestion performance improvement and microbial characteristics. Furthermore, the negative impacts of conductive materials on downstream processing, ecological environment, and anaerobic digestion performance restrict the practical application of DIET (Nguyen et al., 2021). Therefore, a review of literatures on mechanisms, performances, and challenges of DIET improvement *via* adding conductive materials in anaerobic digestion are imperative for a better vision in the practical application.

This review comprehensively summarizes the deep-rooted mechanisms of DIET and systematically explores the advantages and effects of DIET mediated by conductive materials in anaerobic digestion systems. Furthermore, this review focuses on existing challenges and critically discusses the unclear biological mechanisms of DIET, influences of non-DIET mechanisms, limitations of organic matters syntrophically oxidized by way of DIET, and problems in practical application of DIET mediated by conductive materials. Finally, the future research directions of DIET proposed herein are expected to provide a theoretical basis and reference for practical application.

## Establishment of Direct Interspecies Electron Transfer in Anaerobic Digestion

**FIGURE 1:**
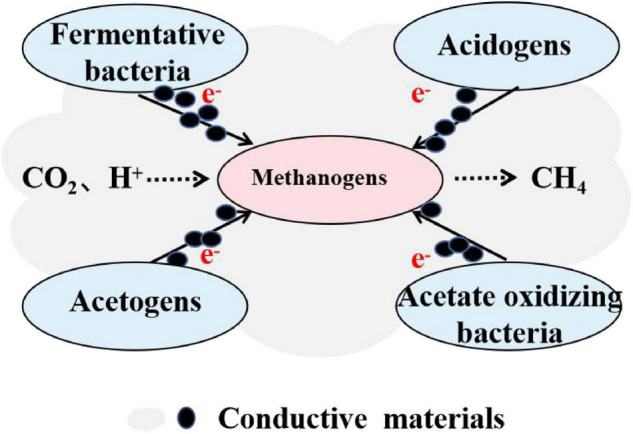
Possible mechanisms on establishing direct interspecies electron transfer (DIET) *via* addition of conductive materials.

Recently, the establishment of DIET by adding conductive materials to promote anaerobic digestion performance has become a hot topic. The realization of DIET methanogenic pathway no longer entirely rely on biological tools such as e-pili or OmcS because of the presence of conductive materials (Chen et al., 2014b; Xu et al., 2019). Further, the addition of conductive materials enriches microorganisms with an extracellular electron transfer ability (Nguyen et al., 2021). It can be speculated that these microorganisms may participate in different stages of anaerobic digestion (Figure 1), making it possible to develop the DIET pathway in methanogenic systems with different substrates (Yin and Wu, 2019; Li et al., 2021). Therefore, many studies have focused on the establishment of DIET in anaerobic digestion reactors by adding different types of conductive materials.

Another promising strategy is to initially provide ethanol stimulation to anaerobic digestion (Zhao et al., 2015, 2017a; Li et al., 2020b). This method achieves the purpose of enriching *Geobacter* and overcomes the lack of *Geobacter* in most traditional methanogenic communities (Zhao et al., 2016b). Its possible mechanism may be that the energy produced by ethanol metabolism can promote DIET to overcome the thermodynamic limitation of syntrophic oxidation (Zhao and Zhang, 2019). Based on these advantages, the ethanol-type fermentation product, which is used as a substrate, will continuously provoke the methanogenic communities to perform DIET, avoiding the economic problems related to the extra supply of ethanol (Zhao et al., 2017a). However, this coupling process is complex and increases the operation cost. Besides, not all organic wastes are suitable substrates for ethanol fermentation.

Studies have found that even if the substrates contain ethanol, the addition of conductive materials can further stimulate the DIET and promote anaerobic digestion performance (Yang et al., 2015a; Zhao et al., 2016b; Li et al., 2020a). This may be because that the DIET mediated by conductive materials can realize long-distance electron transfer (Martins et al., 2018), thereby making the electron transfer more efficient compared to the ethanol stimulation. Furthermore, conductive materials have the advantages of being readily available and relatively inexpensive. Therefore, establishing DIET by adding conductive materials remains an attractive option.

## Direct Interspecies Electron Transfer Mechanisms

### Biological Structure Mediating Direct Interspecies Electron Transfer

**FIGURE 2:**
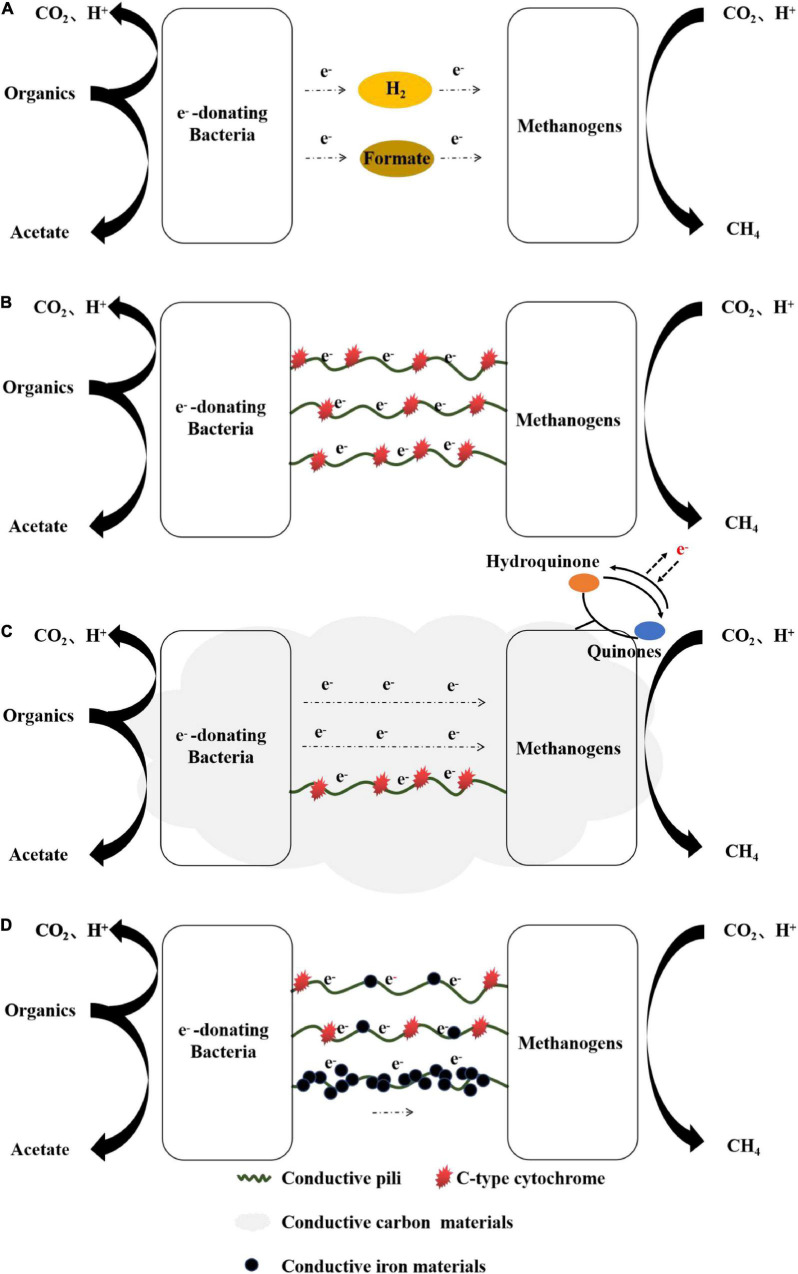
Electron transfer mechanisms **(A)** IHT and IFT, **(B)** biological structure mediating DIET, **(C)** conductive carbon material mediating DIET, and **(D)** conductive iron material mediating DIET.

Generally, researchers conduct in-depth research on the mechanisms of DIET in defined co-culture systems using ethanol as a substrate (Table 1). Compared with indirect electron transfer, which relies on H_2_ and formate as electron carriers (Figure 2A), biological structure mediating DIET achieves electrical connection *via* e-pili and OmcS of electron-donating microorganisms (Summers et al., 2010). E-pili, also known as nanowires, are protein filaments produced by microorganisms during electron transfer under appropriate conditions (Malvankar and Lovley, 2012). Malvankar et al. (2011) found that the conductivity of pili protein extracted from *Geobacter sulfurreducens* was approximately 5 μS/cm, and exhibited temperature dependence similar to that of disordered metals. OmcS is a type of Fe(III) redox protein that is related to the process of electron transfer from cell surface to extracellular electron acceptor. Leang et al. (2010) observed that the OmcS was mainly distributed along nanowires or exists on the outer surface of cells using scanning electron microscopy. Shrestha et al. (2013) found that the key genes encoding e-pili and OmcS were highly expressed in the co-culture system of *G eobacter metallireducens* and *G. sulfurreducens*. Rotaru et al. (2014b) confirmed that co-culture of microorganisms cannot reproduce without the key genes encoding e-pili and OmcS. Moreover, e-pili- or OmcS-deficient strains cannot metabolize ethanol to produce methane in the defined co-cultures (Rotaru et al., 2014a,b). These results indicate that e-pili and OmcS play an important role in establishing DIET. The possible related mechanisms are summarized in Figure 2B.

**TABLE 1:** Summary of studies on direct interspecies electron transfer (DIET) in defined co-culture.

Defined co-culture	Electron donor	Electron acceptor	Mediator	References
*G. metallireducens* and *M. barkeri*	Ethanol	CO_2_	Activated carbon, e-pili and OmcS	Liu et al., 2012
*G. metallireducens* and *M. harundinacea*	Ethanol	CO_2_	E-pili and OmcS	Rotaru et al., 2014b
*G. metallireducens* and *M. barkeri*	Ethanol	CO_2_	GAC, e-pili and OmcS	Rotaru et al., 2014a
*G. metallireducens* and *G. sulfurreducens*	Ethanol	Fumarate	Carbon cloth, e-pili and OmcS	Chen et al., 2014a
*G. metallireducens* and *G. sulfurreducens*	Ethanol	Fumarate	Biochar, e-pili and OmcS	Chen et al., 2014b
*G. metallireducens* and *G. sulfurreducens*	Ethanol	Fumarate	Magnetite, e-pili and OmcS	Liu et al., 2015
*G. metallireducens* and *M. barkeri*	Ethanol	CO_2_	E-pili and OmcS	Holmes et al., 2018
*G. metallireducens* and *G. sulfurreducens*	Ethanol	Fumarate	OmcS	Liu et al., 2021

The mechanism of methanogens to accept electrons may be similar to that of electron donation microorganisms (Li et al., 2021). However, methanogens, including *Methanosarcina barkeri*, and *Methanosarcina harundinaceae* have been confirmed to participate in DIET without OmcS on their outer surface (Holmes et al., 2018; Yee and Rotaru, 2020). In addition, Walker et al. (2019) reported *Methanospirillum hungatei* could secrete e-pili. However, *M. hungatei* could not conduct the DIET with *G. metallireducens* in the co-culture system (Rotaru et al., 2014b). The study on extracellular electron transfer mechanism of methanogens has just begun, which is far less than the understanding with *Geobacter* species. Therefore, it is necessary to deeply study the molecular targets related to biological pathway of methanogens to accept electrons.

### Conductive Carbon Material Mediating Direct Interspecies Electron Transfer

Conductive carbon materials have been proven to significantly improve anaerobic digestion performance (Lee et al., 2016; Lin et al., 2017; Li J. et al., 2018). Liu et al. (2012) thought that the reason why active carbon promoted DIET was that the conductivity of active carbon was higher than that of methanogenic aggregates. However, the promotion effect of biochar is almost the same as that of active carbon even though the conductivity of biochar is one-thousandth that of activated carbon (Chen et al., 2014b). Therefore, it can be inferred that any conductivity greater than a certain threshold is sufficient to trigger the DIET methanogenic pathway (Barua and Dhar, 2017).

Direct interspecies electron transfer promoted by conductive carbon materials is not completely dependent on e-pili or OmcS as the media for electron transfer (Zhao and Zhang, 2019). Rather, the electrical connection was realized through carbon materials, not intercellular contact, because there were no aggregates of *G. metallireducens* and *M. harundinaceae* on the surface of carbon materials (Chen et al., 2014b). Liu et al. (2012) confirmed that e-pili- and OmcS-deficient strains could metabolize ethanol and reduce fumarate to succinate with addition of conductive carbon materials. Park et al. (2018b) found that conductive carbon materials made the abundance of key genes encoding e-pili and OmcS in wild-type strains decrease compared with the control, indicating that conductive carbon materials replaced the functions of e-pili and OmcS. Therefore, establishing DIET by adding conductive carbon materials might consume less energy to satisfy the growth of syntrophic partners and metabolize more substrates as the microorganisms did not need to highly express the key genes encoding e-pili and OmcS (Zhao et al., 2015).

Furthermore, conductive carbon materials have large specific surface areas and porosities (Figueiredo et al., 1999; Chen et al., 2014b; Zhao et al., 2016a), which play a vital role in the adsorption, fixation, and enrichment of microorganisms involved in DIET (Qi et al., 2021). Meanwhile, the redox groups on the surface of conductive carbon materials, such as quinone or quinone hydrogen, may be closely related to DIET (Wang et al., 2020b). The possible related mechanisms are shown in Figure 2C.

### Conductive Iron Material Mediating Direct Interspecies Electron Transfer

Conductive iron materials also promote DIET formation (Kato et al., 2012; Zhu et al., 2017). Currently, researches are mainly focused on the promotion of DIET by magnetite. Ueki et al. (2018) found that magnetite could not replace conductive carbon materials as adding magnetite did not promote DIET in a co-culture system of e-pili-deficient strains. However, Liu et al. (2015) found that magnetite enabled OmcS-deficient strains to have the ability to realize extracellular electron transfer. They found that the expression of wild-type OmcS gene was inhibited because of the presence of magnetite. Moreover, transmission electron microscopy showed that magnetite, similar to OmcS, was dispersed between cells and distributed along the e-pili (Liu et al., 2015). These results indicated that magnetite replaced the role of OmcS rather than that of e-pili (Lovley, 2017). The stimulating effect of magnetite is different from that of conductive carbon materials probably because of their size and structural differences. Magnetite particles are generally 20–50 nm in size, which is smaller than microbial cells, while conductive carbon materials have a significantly bigger size and provide a large surface area for microbial attachment (Lovley, 2017; Yin and Wu, 2019; Zhang et al., 2020).

In addition, many studies have shown that conductive iron materials can enrich microorganisms with extracellular electron transfer abilities, which may be involved in DIET (Yamada et al., 2015; Yang et al., 2015b). The possible related mechanisms are shown in Figure 2D.

## Improvements of Anaerobic Digestion Performances

The establishment of DIET-based syntrophic relationships *via* adding conductive materials improve anaerobic digestion performance in several aspects under different working conditions (Table 2).

**TABLE 2:** Summary of studies regarding enhancement effect of DIET in anaerobic digestion systems.

Material type	Dose	Particle size	Substrate	Strengthening effect	Section	References
Biochar	10 g/L	75 μm	Glucose	Lag phase decreased by 38.0%.	Shortening Lag Period and Start-Up Time	Luo et al., 2015
Carbon nanotube	1 g/L	1–2 nm	Glucose	Start-up period shortened by around 40%.	Shortening Lag Period and Start-Up Time	Yan et al., 2017
Biochar	15 g/L	0.25–1 mm	Phenol	Lag phase decreased from 15.0 days to 1.1–3.2 days	Shortening Lag Period and Start-Up Time	Wang et al., 2020a
Hematite or magnetite	25 mmol/L Fe	–	Benzoate	Lag phase shortened by 8–12 days	Shortening Lag Period and Start-Up Time	Zhuang et al., 2015
Biochar	2–15g/L	0.25–1 mm	Activated and food waste	Lag time decreased by 27.5–64.4%.	Shortening Lag Period and Start-Up Time	Wang P. et al., 2018a
Fe_3_O_4_	10 g/L	–	Synthetic wastewater	Lag time decreased by 13.9%.	Shortening Lag Period and Start-Up Time	Yin et al., 2017b
Biochar	20 g/L	–	VFAs	Lag phase shortened by 9.1–29.2%.	Shortening Lag Period and Start-Up Time	Wang et al., 2020b
GAC	6 g/L	<100 μm	Acetic acid and ethanol	CH_4_ yield increased by 31%.	Improving Methane Yield	Park et al., 2018b
GAC	–	8–20 mesh	Lipid-rapeseed oil	CH_4_ yield increased by 3.9 times.	Improving Methane Yield	Zhang et al., 2020
Biochar	10 g/L	10–15 mm	Kitchen wastes and waste sludge	CH_4_ yield increased by about 44%.	Improving Methane Yield	Li et al., 2020a
Biochar	0.5–1.5 g/g⋅VS	100 mesh	Food waste and sewage sludge	Lag time of CH_4_ production decreased.	Improving Methane Yield	Jiang et al., 2020
Activated carbon	2–12 g/L	180–200 mesh	Sewage sludge	CH_4_ yields improved by 124.0–146.3%.	Improving Methane Yield	Sun et al., 2020
Graphene	30 and 120 mg/L	Nano	Glucose	CH_4_ yields increased by 17.0 and 51.4%, respectively.	Improving Methane Yield	Tian et al., 2017
Carbon-based materials	–	–	Dog food	Higher organic loading rates were permitted.	Enhancing System Stability	Dang et al., 2016
Carbon-based materials	–	–	Municipal solid waste	CH_4_ production was promoted under high VFAs concentrations.	Enhancing System Stability	Dang et al., 2017
Biochar	10 g/L	2–5 mm	Glucose	Lag phase shortened and CH_4_ yields were improved.	Enhancing System Stability	Lu et al., 2016
Magnetite	25 mmol/L Fe	50–100 nm	Pig manure	CH_4_ yields increased with high ammonia concentrations.	Enhancing System Stability	Zhuang et al., 2018
Magnetite	20 mmol/L Fe	1.2 ± 0.2 μm	Artificial wastewater	CH_4_ production was increased 3–10 folds.	Enhancing System Stability	Jin et al., 2019
Stainless steel	25.7 g/L	0.5–2 mm	Artificial wastewater	CH_4_ production increased by 7.5–24.6%.	Enhancing System Stability	Li et al., 2017

### Shortening Lag Period and Start-Up Time

Lag period is the time required for inoculum to adapt to the new environment. A long lag period means that a longer start-up time is needed for anaerobic digestion. In a practical start-up process, microorganisms must firstly recover from dormancy. The growth rate of methanogens in the original anaerobic sludge is much slower than that of bacteria. Usually, anaerobic digestion reactors start with short hydraulic retention time, low organic load rate (OLR), and low feeding concentration. These factors lead to a longer start-up cycle, which affects anaerobic digestion efficiency and causes an increase in operating cost. Studies have shown that DIET mediated by conductive materials can significantly shorten lag time of anaerobic digestion reactor with different types of substrates, such as glucose (Luo et al., 2015; Yan et al., 2017), phenol (Wang et al., 2020a), benzoate (Zhuang et al., 2015), and mixed organic carbon (Yin et al., 2017b; Wang G. et al., 2018). Moreover, establishing DIET by adding conductive materials significantly shortened the lag period of anaerobic digestion system even under conditions of high hydrogen partial pressure, high concentration of ammonia nitrogen, and sulfur inhibition. Wang et al. (2020b) found that DIET stimulation shortened the lag time of acetate, propionate, butyrate, and valerate by 29.2, 20.5, 9.1, and 10.5%, respectively. Therefore, the promotion on syntrophic metabolism of volatile fatty acids (VFAs) may be the main reason why DIET shortens the lag period.

### Improving Methane Yield

As DIET is more efficient than IHT, the establishment of DIET mediated by conductive materials has been widely recognized to promote methane yield. Park et al. (2018b) found that methane yield with GAC addition increased by 31% compared to control without GAC addition. Similarly, Zhang et al. (2020) observed that the methane yield in GAC-amended systems increased by 3.9 times. Li et al. (2020a) reported that the methane yield with biochar increased by about 44% and the effluent concentration of total organic substrate decreased compared with control. Besides, improvement in the methane yield has been also repeatedly reported and verified with other conductive materials, such as graphite (Tan et al., 2015), magnetite (Ye et al., 2018), and carbon cloth (Zhao et al., 2017c). Studies showed that the increase in methane yield is mainly because that DITE mediated by conductive materials facilitated the conversion of interspecies metabolites to methane (Tian et al., 2017; Jiang et al., 2020; Sun et al., 2020).

### Enhancing System Stability

Direct interspecies electron transfer mediated by conductive materials plays an important role in maintaining the stability of anaerobic digestion system. To achieve efficient energy production, anaerobic digestion systems are generally maintained at high OLRs (Appels et al., 2011). However, high OLRs often cause the acidogenesis rate to be faster than methanogenesis rate, resulting in a sharp drop in pH and VFA accumulation (Babel et al., 2004). Nevertheless, DIET can still improve anaerobic digestion performances under high OLR conditions (Dang et al., 2016, 2017). Moreover, a high hydrogen partial pressure is another factor that leads to VFA accumulation. However, DIET-based syntrophic relationships can enable anaerobic digestion systems to overcome the limitation of hydrogen partial pressure (Nagarajan et al., 2013; Zhao et al., 2016b; Zhao Z. et al., 2018). Organic matters containing nitrogen (protein, amino acids, and urea) can release high concentration of ammonia in an anaerobic digestion system. The ammonia can exist in the form of ammonium ion or free ammonia, which inhibit microbial activity and cell growth (Kayhanian, 1999). However, Lu et al. (2016) found that the methane yield increased with the addition of biochar under an ammonia inhibition environment. This may be because that the establishment of DIET resisted the toxic inhibition of ammonia and maintained the stability of syntrophic metabolism. In addition, supplemental magnetite significantly increased the conversion rate of acetate to methane under high ammonium concentrations (Zhuang et al., 2018). Sulfate in the anaerobic digestion system can stimulate the growth of sulfate-reducing bacteria which compete with methanogens for acetate, H_2_, and intermediates, such as propionate, butyrate, and ethanol (Li Q. et al., 2015; Qian et al., 2019). Hydrogen sulfide, the product of sulfate reduction, can significantly inhibit the activities of methanogens, which decreases the anaerobic digestion performances (Chen et al., 2008). However, some studies have demonstrated that DIET mediated by conductive materials was expected to promote methane production in anaerobic digestion system treating organic wastewater with a high concentrations of sulfate (Li et al., 2017; Jin et al., 2019). The potential mechanism may be that methanogens through DIET metabolic pathway are more competitive to substrates than sulfate-reducing bacteria (Liu et al., 2019).

## Challenges of Direct Interspecies Electron Transfer in Anaerobic Digestion

Although the establishment of DIET *via* addition of conductive materials shows a significant promotion effect in anaerobic digestion system, the current research is only in its primary stage. Therefore, this section discusses the existing challenges.

### Unclear Biological Mechanism of Direct Interspecies Electron Transfer

**FIGURE 3:**
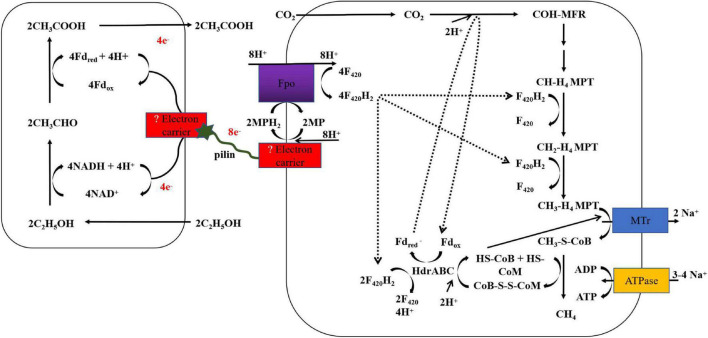
DIET model in co-culture of *G. metallireducens* and *M. barkeri.* This figure is adapted from previous paper (Rotaru et al., 2014a; Holmes et al., 2018).

Study on the biological pathway of DIET is helpful for understanding microbial syntrophic metabolism. Figure 3 shows the model of DIET methanogenic pathways between *G. metallireducens* and *M. barkeri*. However, the electron transfer biological pathway in DIET between microorganisms remains unclear.

To date, it is unclear how electron donating bacteria transfer electrons outside of the cell. *Geobacter* is often used as a model microorganism to study the mechanism of extracellular electron transfer because of its completely available genome sequence and skilled gene engineering methods (Coppi et al., 2001). In general, it is believed that the e-pili of *Geobacter* transfer electrons to OmcS and then to extracellular insoluble electron acceptors such as iron oxides and electrodes. However, the mechanism of electron transfer to extracellular e-pili is unclear. Currently, cytochromes (OmaB, OmaC, OmcB, and OmcC) and porins (OmbB and OmbC) are considered to play essential roles in the transfer of electrons to e-pili (Costa et al., 2018). Besides, it remains unclear how electron-accepting microorganisms directly accept extracellular electrons from their syntrophic partners. Studies on extracellular electron transfer showed that OmcS and pili nanowires were indispensable during the process of extracellular electron transfer. However, unlike *Geobacter* species, *M. barkeri*, and *M. harundinaceae* without outer surface e-pili and OmcS have also been found to be involved in DIET (Holmes et al., 2018). Therefore, it is necessary to further find the differential expression of genes in electroactive methanogens under different methanogenic conditions, and then verify the functions of related genes through gene mutations, and finally reveal the extracellular electron transfer mechanism of electron-donating microorganisms. Finally, it is unclear how methanogens utilize extracellular electrons and reduce CO_2_ through DIET. Thauer et al. (2008) proposed that the CO_2_ reduction pathways of methanogens with and without cytochromes might vary. Recent research showed that DIET promoted transcripts of F_420_H_2_ dehydrogenase (Fpo) and heterodisulfide reductase (HdrABC) (Holmes et al., 2018). Electrons transferred *via* the DIET pathway were first employed to reduce methanophenazine (MP), and then the generated MPH_2_ could be used as the electron donor for Fpo to reduce coenzyme F_420_. Half of the produced F_420_H_2_ in this model was regarded as the electron donor for HdrABC bifurcation reaction to further yield reduced ferredoxin for CO_2_ reduction (Holmes et al., 2018). Simultaneously, a bifurcation reaction facilitated the reduction of coenzyme B-coenzyme M heterodisulfide (CoB-S-S-CoM), which is necessary for CH_4_ production. However, how electrons are transferred to MP should be further studied (Holmes et al., 2018).

Recently, Holmes et al. (2021) proposed a new model of DIET methanogenic pathways, which was confirmed in the defined co-culture of *G. metallireducens* and *Methanosarcina acetivorans*. Multiheme membrane-bound c-type cytochrome A (MmcA) in *M. acetivorans* played an important role in electron transfer. GAC did not make the co-culture system with MmcA-deficient strains produce methane rapidly, which was mainly because that MmcA was embedded in the cell membrane of *M. acetivorans* (Ferry, 2020). Researches show that MmcA may exchange electrons with MP or rhodobacter nitrogen (Rnf) complex (Suharti et al., 2014; Ferry, 2020; Holmes et al., 2021). The function of the Rnf is to accept electrons to produce the reduced ferredoxin, which is required for the first step in the reduction of CO_2_ (Holmes et al., 2021). The model in *M. acetivorans* is significantly different from that in *M. barkeri*, because *M. Barkeri* lack genes for MmcA and Rnf (Zhou et al., 2021). Therefore, Methanogen growth *via* DIET may adopt different electron-acceptance strategies to accommodate these physiological differences (Holmes et al., 2021).

### Influence of Non-direct Interspecies Electron Transfer Mechanisms

The positive effect of conductive materials on anaerobic digestion performances has been confirmed. Therefore, most studies speculated that performance improvement might be attributed to DIET existing in complex anaerobic digestion systems based on microorganisms already known to take part in DIET. However, the relative abundance of *Geobacter* in anaerobic sludge is low (Dang et al., 2016; Peng et al., 2018; Li et al., 2020b) or even undetectable (Wang et al., 2020b). In addition, several studies have demonstrated that conductive materials would enrich microorganisms with extracellular electron transfer ability, such as *Desulfuromonas* (Barua et al., 2018), *Pseudomonas* (Barua and Dhar, 2017), *Azovibrio* (Ruiz et al., 2014), and *Syntrophomonas* (Chen Y. et al., 2020), which may participate in DIET. However, it has not been proven that these microorganisms can conduct DIET in defined co-cultures with methanogens. Therefore, it is insufficient to infer that DIET occurred in anaerobic digestion systems based on the anaerobic digestion performance improvement and microbial community structure.

**FIGURE 4:**
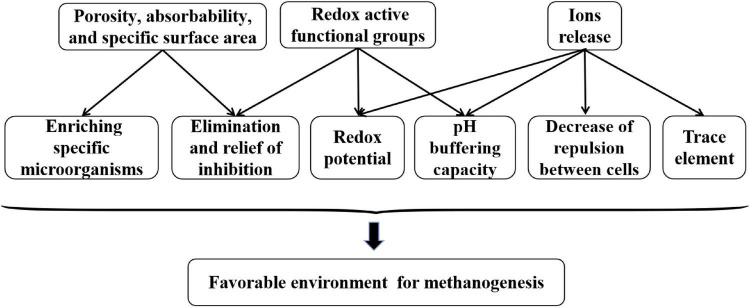
Enhancement effects of non-DIET mechanisms.

In addition to DIET, other stimulatory mechanisms of conductive materials in anaerobic digestion cannot be ignored. The related mechanisms are illustrated in Figure 4. Conductive carbon materials not only promote metabolism of inhibitory intermediates by fixing and enriching specific microorganisms but also alleviate inhibition of toxic substances *via* adsorption effect (Martins et al., 2018; Jung et al., 2020). The pH buffering capacity of conductive carbon materials also creates a favorable environment for methanogenesis (Wang et al., 2017, 2020b). Simultaneously, conductive carbon materials can improve anaerobic digestion performances through other mechanisms, such as function of released ions and surface redox groups, which may affect the redox potential of anaerobic digestion systems. In general, it is believed that a redox potential between −200 and −400 mV is beneficial for methane production (Hirano et al., 2013). Salvador et al. (2017) confirmed that a more negative redox potential occurred with an increase in the carbon nanotube dosage in co-culture systems. Similarly, the effects of non-DIET mechanisms cannot be neglected with addition of conductive iron materials. The iron ions released from conductive iron materials can decrease the negative charge on the surface of extracellular polymeric substances, thereby reducing the repulsive force between cells and increasing the stability of microbial aggregates. Furthermore, the release of iron ions might be regarded as a trace element that promotes microorganism activity.

Therefore, whether the improvement of anaerobic digestion performances is caused by DIET mediated by conductive materials and the DIET is the dominant mechanism in a complex anaerobic digestion system requires in-depth exploration and analysis.

### Limitations of Organic Matters Syntrophically Oxidized by Way of Direct Interspecies Electron Transfer

Ethanol has been proven to participate in DIET methanogenesis in a defined co-culture (Rotaru et al., 2014a,b). Although acetate has not been proven to be a substrate that participates in DIET, Zhuang et al. (2018) found that the degradation of acetate in bioreactor containing magnetite was much less affected by hydrogen partial pressure, which may be because that the acetate oxidation metabolism was not completely dependent on IHT. Propionate and butyrate are important intermediate metabolites in anaerobic digestion. Thus, limiting propionate and butyrate accumulation is crucial for improving anaerobic digestion performances. Studies showed that magnetite significantly increased propionate and butyrate degradation, suggesting that propionate and butyrate may be converted to methane *via* DIET metabolic pathway (Cruz Viggi et al., 2014; Li H. et al., 2015; Jing et al., 2017). Similarly, Barua et al. (2018) confirmed that carbon fibers promoted the conversion of propionate and butyrate to methane. In a syntrophic propionate metabolism, Cruz Viggi et al. (2014) confirmed that conductive materials provided 10^6^ times higher electron carrier flux than that of IHT. In a syntrophic butyrate metabolism, Xu et al. (2018) found that the conversion rate of butyrate into acetate increased by 2.5–7.0 times when conductive material dosage increased from 0.5 to 25 g/L, which may be because that the increase in conductive material dosage provided more carriers for syntrophic partners to exchange electron (Yin and Wu, 2019). Zhuang et al. (2015) found that the addition of magnetite and hematite promoted the anaerobic digestion performances of benzoate. Similar studies demonstrated that adding magnetite or carbon nanotubes to an anaerobic digestion system accelerated the degradation of phenol and reduced its inhibitory effect on biological activity of methanogens (Yan et al., 2018). In addition, glucose and sucrose have been widely reported as substrates of DIET methanogenesis. However, in addition to ethanol as a substrate that participates in DIET, the establishment of DIET metabolic pathway in an anaerobic digestion system with other organics as substrates lacks direct biological evidence.

Compared with simple organics, complex organics must undergo hydrolysis and acidification before methanogenesis. Therefore, the DIET mechanisms of complex organics may be more complicated than that of simple organics. Yang et al. (2017) and Sun et al. (2020) confirmed that addition of conductive materials improved the conversion rate of sludge to methane. Similarly, although the anaerobic digestion of leachate from municipal solid waste is limited by high OLRs and toxic substances, some studies found that establishing DIET could increase the digestibility of leachate and methane production (Lei et al., 2016, 2018). However, Zhao et al. (2017b) added magnetite and GAC to hydrolysis-acidogenesis and methanogenesis reactors, respectively, and found that the supplemental GAC had little effect on syntrophic metabolism of alcohols and VFAs when hydrolysis-acidogenesis was ineffective or did not work. After magnetite promoted acidogenesis efficiency, the syntrophic metabolism of alcohols and VFAs in methanogenesis phase was markedly enhanced by GAC. Anaerobic digestion modified by magnetite can enrich iron-reducing bacteria, which play an important role in the hydrolysis-acidogenesis of complex organics (Peng et al., 2018). The increase in methane production in anaerobic digestion with GAC is probably caused by DIET between syntrophic bacteria and methanogens (Peng et al., 2018). Therefore, establishing DIET may not change the rate-limiting step of anaerobic digestion with complex organic matters because the confirmed DIET mainly occurs in the methanogenic stage. Thus, further researches are necessary to promote the hydrolysis and acidogenesis of complex organics *via* DIET metabolic pathway.

### Problems in Practical Application

There are few successful practical application of DIET in anaerobic digestion *via* adding conductive materials. This is mainly because that the establishment of DIET does not achieve satisfactory results in amplification experiments and demonstration projects. The complexity of reactor operation control makes it difficult to ensure that conductive materials take effect in the engineered anaerobic digestion system. In addition to considering the positive effect of DIET mediated by conductive materials, it is also important to consider the economic cost and benefit. Although researchers usually add conductive materials to anaerobic digestion, this method is not economically scalable (Zhao et al., 2020). There is loss of conductive materials in the digester discharge (Yin et al., 2017a; Zhang et al., 2018). For example, Zhang et al. (2018) found that the cost required to maintain the level of GAC in the anaerobic digestion accounted for 38.3–81.8% of the revenue from methane production each day. The labor for supplement of conductive materials also increases the economic cost on a daily basis (Zhao et al., 2020). Furthermore, the impacts of conductive material discharge on ecological environment and downstream processing should be valued. Currently, there are few studies on the influence of adding conductive materials to anaerobic digestion system on sludge dewatering (Nguyen et al., 2021). Obviously, addition of conductive materials significantly increases the amount of biosolids (Chen S. et al., 2020; Yuan et al., 2021), which increases the cost of solid waste transportation and treatment in the later stage. Recycling and reuse of conductive materials may be an effective strategy, but the complex technical operation and high investment cannot be ignored. Some studies have suggested that conductive materials designed as packed beds inside the reactor should be considered to continually promote DIET. However, this approach usually incurs considerable cost for the maintenance of equipment and construction of reactor (Tatara et al., 2008; Zhao and Zhang, 2019).

Some researchers have also found that conductive materials may have an inhibitory effect on anaerobic digestion performances. For example, Tian et al. (2017) found that the methane yield at a graphene dosage of 30 mg/L was 14.3% higher than that of the control after long-term operation at room temperature, while the methane yield at a graphene dosage of 120 mg/L exhibited a slight inhibitory effect. Salvador et al. (2017) found that carbon nanotubes of 5 g/L inhibited methane production compared with dosages of 0.5 and 1 g/L. These results indicated that excessive dosage of conductive materials could inhibit the microbial metabolism. The potential inhibitory effect may be attributed to the increased resistance to mass transfer between substrates and microorganisms. In addition, studies showed that the supplementation of carbon black nanoparticles resulted in a significant inhibitory effect on an anaerobic digestion system treating glucose, which might be because that carbon black nanoparticles possessed antibacterial properties (Martins et al., 2018; Fujinawa et al., 2019). Similar to conductive carbon materials, conductive iron materials, such as ferrihydrite, also exhibit an inhibitory effect on methane production (Zhou et al., 2014; Martins et al., 2018). Therefore, the dosage and type of conductive material may have unpredictable effect on the anaerobic digestion system. Further, the surface structure of conductive materials, such as the specific surface area, pore size, and roughness, affects the adhesion and colonization of relevant microorganisms in anaerobic digestion process. These factors limit the practical application of DIET mediated by conductive materials in anaerobic digestion.

## Conclusion and Perspectives

The DIET has been proven to be more efficient than IHT in terms of electron transfer efficiency and energy conservation. Therefore, the establishment of DIET by adding conductive materials can significantly enhance anaerobic digestion performances, and have attracted increasing attention in recent years. This review summarizes the DIET mechanisms and promotion effect of DIET mediated by conductive materials on anaerobic digestion system. Based on our understanding, this review presents current challenges including unclear biological mechanism of DIET, influences of non-DIET mechanisms of conductive materials, limitations of organic matters syntrophically oxidized by way of DIET, and problems in practical application of DIET mediated by conductive materials. Therefore, more detailed exploration researches are necessary to fill up the gaps of relevant knowledge.

Limited by biological means and technical level, it is difficult to track electron transfer paths throughout the DIET process and determine all proteins and structures involved. Therefore, further researches are necessary to analyze the biological mechanisms of DIET in order to provide a theoretical reference for the application of DIET mediated by conductive materials in anaerobic digestion. As the knowledge regarding DIET enhancement by conductive materials is limited, the current researches only speculated that DIET occurred in the anaerobic digestion based on the following phenomena: (1) the microbial aggregates exhibited metal-like electrical conductivity; (2) *Geobacter*, *Methanosaeta*, and *Methanosarcina* were dominant in the microbial community structure; and (3) the anaerobic digestion system could overcome the limitation of hydrogen partial pressure. However, these studies did not provide direct evidence for these conclusions. Therefore, the interaction mechanisms between conductive materials and microorganisms require further exploration. The metabolic network of complex organic matters in anaerobic digestion is intricate. Therefore, future researches are also necessary to discover more microorganisms capable of DIET, which provides the possibility of complex organic matters that participate DIET metabolic pathway. Moreover, interdisciplinary development may be beneficial for the application of DIET in anaerobic digestion as the establishment of DIET mediated by conductive materials involves the integration of microbiology, biochemistry, material science, and other subjects.

## Author Contributions

LC conceptualized and drafted the manuscript. PZ revised the manuscript and supervised the study. WF and GZ improved the manuscript. JC and JL revised the manuscript. All authors approved the submitted version.

## Conflict of Interest

The authors declare that the research was conducted in the absence of any commercial or financial relationships that could be construed as a potential conflict of interest.

## Publisher’s Note

All claims expressed in this article are solely those of the authors and do not necessarily represent those of their affiliated organizations, or those of the publisher, the editors and the reviewers. Any product that may be evaluated in this article, or claim that may be made by its manufacturer, is not guaranteed or endorsed by the publisher.
